# Dietary energy level modulates cecal microbiota to influence amino acid composition of local chicken meat

**DOI:** 10.1128/spectrum.02046-25

**Published:** 2026-03-23

**Authors:** Jingjing Li, Yidan Xu, Qingke Huang, Lili Xian, Lingqian Yin, Yiping Liu, Jing Feng

**Affiliations:** 1 College of Life Science and Agri-forestry, Southwest University of Science and Technology 91609, Mianyang, Sichuan, China; 2 Guangyuan City animal husbandry seed management station, Guangyuan, Sichuan, China; 3 Institute of Animal Husbandry and Veterinary Medicine, XiZang Academy of Agricultural and Animal Husbandry Science 670600 , Lhasa, XiZang, China; 4 Key Laboratory of Animal Genetics and Breeding on Tibetan Plateau, Ministry of Agriculture and Rural Affairs, Lhasa, XiZang, China; 5 Farm Animal Genetic Resources Exploration and Innovation Key Laboratory of Sichuan Province, Sichuan Agricultural University 506176 , Chengdu, Sichuan, China; The University of Tennessee Southern, Pulaski, Tennessee, USA

**Keywords:** amino acids, chicken, correlation analysis, gut microorganisms, metabolomics

## Abstract

This study comprehensively investigated the effects of different dietary energy levels—low energy (LE, 10.62 MJ/kg), medium energy (ME, 11.87 MJ/kg), and high energy (HE, 13.06 MJ/kg)—on growth performance, meat quality, muscle metabolite profiles, and cecal microbiota in a local Chinese chicken breed (Guangyuan gray chicken). Growth and carcass traits, including interpubic width and liver weight, responded positively to increasing dietary energy. Compared to the ME control, the HE diet significantly increased drip loss, whereas the LE diet reduced both intramuscular fat and protein content (*P* <0.05). Untargeted metabolomic analysis identified 188 differentially abundant muscle metabolites across the dietary groups, revealing distinct metabolic profiles. The biosynthesis of the amino acids pathway was significantly affected by different dietary energy levels. Targeted metabolomics further demonstrated the differences in muscle amino acid composition: essential, sweet-taste, and umami-taste amino acids increased progressively with dietary energy, with the HE group achieving the most favorable profile for flavor and nutrition. Cecal microbiota analysis indicated that dietary energy levels significantly altered microbial community structure. Specific bacterial taxa, including *Spirochaetota* and *Bacteroides*, were positively correlated with muscle amino acid concentrations. Our findings demonstrate that dietary energy level modulated the cecal microbiota, which in turn influenced muscle amino acid deposition and overall meat quality. This study provides a comparative multi-omics perspective for optimizing dietary energy strategies in local chicken production to meet varying market demands for meat quality and nutritional value.

**IMPORTANCE:**

This comparative study established a crucial link between dietary energy intake and the final quality of poultry meat through a multi-omics lens. We demonstrate that low, medium, and high dietary energy levels elicit distinct phenotypic, metabolic, and microbial profiles in a local chicken breed. The findings provide a scientific framework for selecting dietary energy strategies tailored to specific market demands: HE for premium quality segments, LE for lean or cost-driven production, and ME for efficient conventional production. We identified specific correlations between the cecal bacteria (*Spirochaetota*, *Bacteroides*) and muscle metabolites (key amino acids). Thus, this work offered the scientific data to optimize meat quality through targeted feeding interventions.

## INTRODUCTION

Chickens serve as an important source of meat for humans. With increasing awareness of healthy living, people are paying more attention to the nutritional and health benefits of their diet (1). Meat quality is a key attribute that influences consumer meat preference (2). Previous studies indicate that dietary energy levels had a variable effect on growth performance, carcass characteristics, and meat quality in rapidly growing broilers (3–5). High metabolizable energy decreased feed intake and exacerbated excessive fat deposition in broilers (5). Low dietary metabolizable energy reduced the amount of abdominal fat in broilers and increased tenderness (3). In Cobb broiler chicks, a higher energy level than the nutritional requirements recommended by broilers had a positive effect on pectoral-free amino acids (6).

Compared with the commercial chicken breeds, slow-growing meat-type chicken breeds have a higher sale price and better meat quality and flavor due to their long growth cycle. Due to different growth rates, fast-growing chicken breeds and slow-growing chicken breeds have different nutrient requirements and mechanisms of digestion and absorption (7). Thus, the difference in response to energy and protein level in the diet between these two breeds may affect the metabolite composition, especially amino acids of muscle (8). Guangyuan gray chicken, a local Chinese chicken breed, has the characteristics of coarse feeding resistance, good stress resistance, strong adaptability, good taste, and high nutritional value (9). However, whether dietary energy levels affect growth performance, carcass characteristics, and meat quality in this Chinese native chicken breed remains unclear. Determination of the optimum dietary energy level for native chicken breeds is of paramount importance to maximize their production performance and meat quality.

Metabolomics is an extension of transcriptomics and proteomics and can more directly and accurately reflect physiological changes in organisms (10). Metabolomics facilitates the comprehensive measurement of many small-molecule metabolites found in animal foods (11). A previous study has used metabolomics to determine the correlations between muscle metabolites and meat quality characteristics (9, 12). Therefore, this technique can be used to perform a complete and detailed analysis of meat chemistry and relevant compounds associated with chicken quality traits.

A growing body of research suggests that diets play a key role in regulating meat quality in broilers, primarily through regulation of the intestinal microbiota-muscle axis. Xu et al. showed that a diverse gut microbiota in broilers is linked to muscle growth and metabolism, with a positive correlation with muscle mass and texture (13). By enhancing beneficial gut microbiota through dietary prebiotics, Lactobacillus, and Bifidobacterium supplementation, broilers exhibited improved nutrient absorption and muscle development, concurrently elevated meat tenderness and juiciness (14, 15). The correlation between the gut microbiota and muscle development offers a viable strategy to increase the quality of broiler meat by specifically manipulating the gut microbiome.

In this study, the growth performance, carcass traits, and meat quality parameters were examined following three different energy-level diets. Nontargeted and targeted metabolomics, and 16S rDNA amplicon sequencing were performed to analyze the effects of different dietary energy levels on the muscle metabolite composition and cecum microorganisms. The correlation analysis allowed us to verify the hypothesis that different dietary energy levels could alter the composition of muscle metabolites, especially amino acids, in local chicken meat to improve meat quality.

## MATERIALS AND METHODS

### Animals and experimental design

In total, 600 1-day-old female Guangyuan gray chickens were floor-reared at Sichuan Tianguan Ecological Agriculture and Animal Husbandry Co., Ltd. (Guangyuan City, Sichuan Province); the environmental temperature and humidity of the rearing facility were strictly controlled. From hatch until 10 weeks of age, all chicks were fed a standard commercial starter-grower diet to ensure uniform health and development prior to the initiation of the experimental dietary treatments. When the chickens were 10 weeks old, 300 chickens with similar body weights were randomly chosen and housed in individual cages (400 mm × 400 mm × 225 mm) at the same height to light. This timing was chosen because the period from 10 weeks onward represents the primary muscle growth and fat deposition phase for slow-growing local chicken breeds, during which nutritional interventions most directly influence muscle development and final meat quality. The chickens were randomly divided into three groups, each consisting of five replicates with 20 chickens in each replicate. Their dietary metabolizable energy levels were at three levels: high energy (13.06 MJ/kg, HE), medium energy (11.87 MJ/kg, ME), and low energy (10.62 MJ/kg, LE). The ME group was fed a basal diet based on corn-soybean meal, and the energy level was the same as that recommended by the Feeding Standard of Meat-type Chicken Breeder (NY/T 33-2004), which was used as the control group. The HE group was the basal diet with 10% more energy than the control (ME), whereas the LE group was the basal diet with 10% less energy than the control (ME). The diet is presented in Table 1. The chickens were used in a formal experiment for 70 days and had access to feed and water *ad libitum* throughout the experimental period.

**TABLE 1:** The formulation of the trial diets fed to chicken

	Diets (% as fed)		
	LE	ME	HE
Ingredients			
Corn	59.00	68.00	66.00
Soybean meal	15.00	15.00	15.00
Maize gluten meal	1.20	3.00	4.00
Wheat bran	18.80	7.00	4.00
Soybean oil	0.00	1.00	5.00
Dicalcium phosphate	2.00	2.00	2.00
Premixes^*a*^	2.50	2.50	2.50
Mountain flour (calcium content 38%)	1.00	1.00	1.00
Nutrient Composition of diets (as fed)			
Metabolizable energy (MJ/kg)	10.62	11.87	13.06
Protein (%)	15.29	15.23	15.23
Calcium (%)	1.06	1.05	1.04
Available P (%)	0.55	0.52	0.51
Crude fat (%)	5.23	6.05	9.76
Lysine (%)	0.68	0.65	0.64
Methionine (%)	0.26	0.27	0.28

^*a*^ Premix included the following per kg of feed: vitamin A ≥390 KIU, vitamin D3 ≥150 KIU, vitamin E ≥1,240, vitamin B ≥185, vitamin B2 ≥260, vitamin B6 ≥160, vitamin B12 ≥1.2, vitamin K3 ≥100, D-biotin ≥12, D-pantothenic acid ≥470, folic acid ≥57, niacin ≥1,660, hydrogenated choline ≥15,000, methionine ≥43,000, Fe ≥2,000, Cu ≥380, Mn ≥3,900, Zn ≥2,800, I ≥25, Se ≥10.LE, low energy; ME, medium energy; and HE, high energy.

### Sample collection

**Fig 1:**
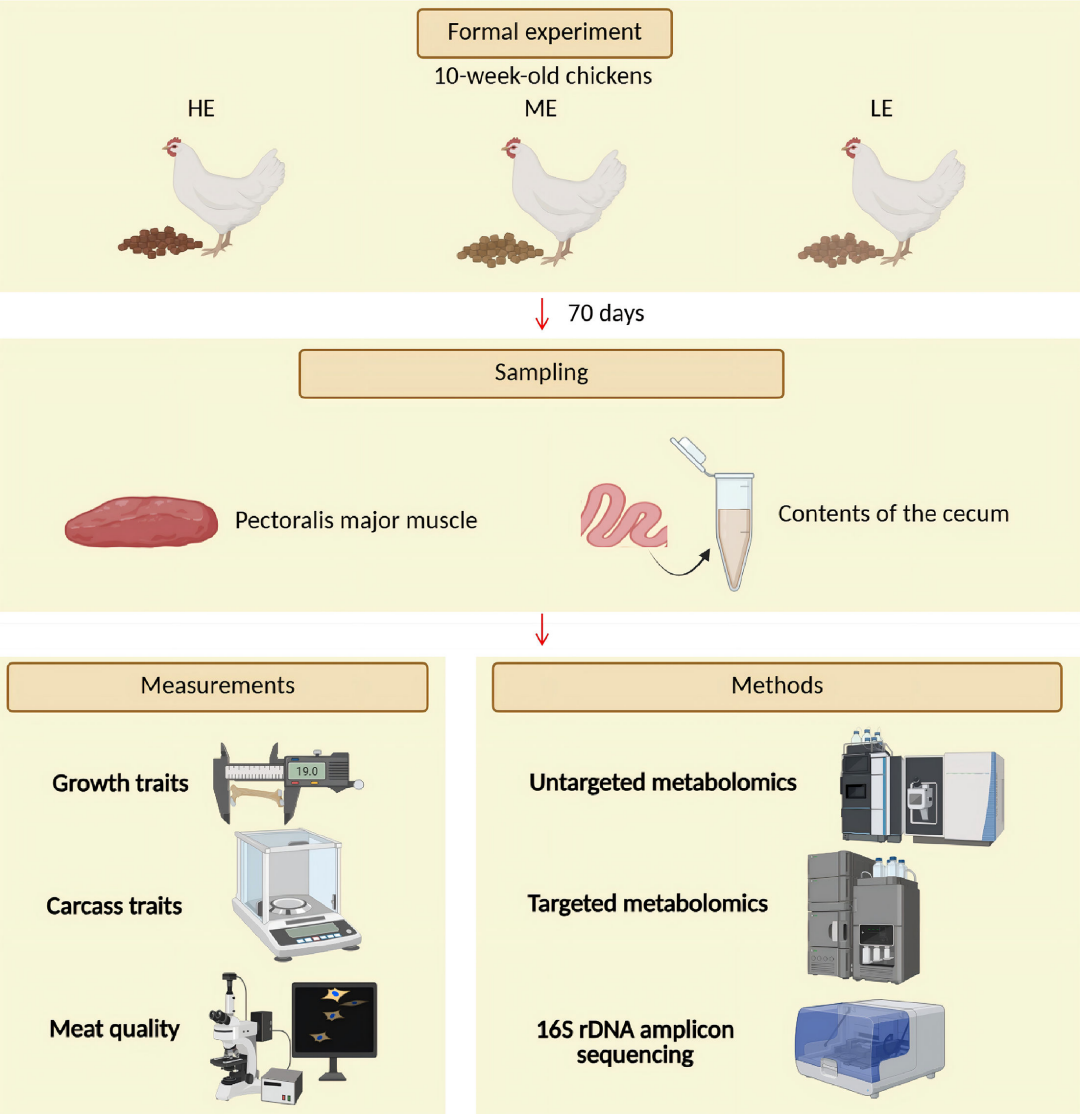
Overview of design and sample collection.

When the chickens were 140 days old, 10 chickens were randomly selected from three treatment groups. They were then slaughtered to measure meat quality, growth, and carcass traits, as well as untargeted and target metabolomics analysis and cecal microbiota analysis (Fig. 1). The pectoralis major muscle was excised. The left pectoralis major muscle was placed in a refrigerator (4°C) to measure meat quality traits, and another meat sample from the right pectoralis major muscle was snap-frozen in liquid nitrogen immediately and stored at –80°C for subsequent analysis. Six replicates per group were analyzed via targeted metabolomics. The contents of the cecum were squeezed out with forceps, immediately placed in a sterile freezer tube, and stored at –80°C. For each group, 10 replicates were analyzed via 16S rDNA amplicon sequencing of the cecal microbiota.

### Growth and carcass traits

When the chickens were 70 days old and 140 days old, 10 chickens were randomly selected from three treatment groups for growth performance assessment. Body weight, body slope length, sternal length, tibia length, tibial circumference, and interpubic width were recorded. When the chickens were 140 days old, they were slaughtered, and the liver weight, pectoral muscle weight, abdominal fat weight, ovarian stroma weight, oviduct weight, and intestinal (duodenum, jejunum, ileum) weight and length were recorded.

### Meat quality parameters

#### Measurement of pH, muscle color, and drip loss

The color was quantified using a Minolta colorimeter (CR-300, Konica Minolta, Japan) based on the CIE system, which measures L∗ (lightness), a∗ (redness), and b∗ (yellowness) values. The final value was also the average of three random readings on the surface of each sample.

Within 1 h of slaughter, 20 mm × 10 mm × 10 mm pieces of the pectoralis major muscle were removed and weighed (W1). The meat sample was tied with muscle fibers pointing downward and hung in an inflatable plastic bag at 4°C for 24 h. The surface juice of each meat sample was wiped off and weighed (W2). The drip loss rate (%) was calculated as [(W1−W2)/W1] *100%.

The pH was measured 24 h after slaughter using a portable pH meter (TEST0205, China). The device penetrated 2 cm into the breast muscle. Measurements were recorded from three areas and averaged (three different points) for each sample.

#### Measurements of moisture, intramuscular fat (IMF), crude protein, and inosine monophosphate (IMP) contents

The contents of moisture, crude fat, and crude protein were determined 48 h after the chickens were slaughtered, following the methods of the Association of Official Analytical Chemists (AOAC) (International, 1995). The moisture content was determined by thoroughly drying the samples in an oven at 103°C ±2°C. The dried sample was ground into a powder and used for further measurement of the IMF content of the sample. Using the Soxhlet extraction method, the IMF content was calculated as [(m1−m0)/m2] *100%, where m2 indicates the sample weight, m1 indicates the weight of the fat and the glass bottle, and m0 indicates the glass bottle weight. The Kjeldahl method was used to determine the protein content of the meat samples. After several sample preparation steps (mincing, homogenizing, centrifuging, and sedimentation), the concentration of IMP in the meat samples was analyzed using high-performance liquid chromatography (HPLC) following the methods described in another study (16).

#### H&E staining and oil red O staining

Hematoxylin and eosin (H&E) staining of the breast muscle was conducted following the methods described in another study (17). Briefly, a 2 cm × 1 cm × 1 cm strip of pectoralis major muscle was cut and soaked in a 4% paraformaldehyde solution for 24 h. Then, the samples were dehydrated with different concentrations of ethanol. The paraffin block was cut into sections (5 μm thick) and stained with H&E solution for 5 min. The sections were sealed with neutral resin. Other muscle tissues were dehydrated with 15% and 30% sucrose solutions. After the samples were placed in OCT embedding medium, a 10-μm paraffin block was cut with a freezing microtome. Next, the samples were fixed with 4% paraformaldehyde for 15 min and stained with Oil Red O (Servicebio, Wuhan, China) for 10 min at room temperature. Images of myofibers were captured under a digital microscope (BA400Digital, Xiamen, China) via an image analyzer (Image‐Pro Plus 6.0) and magnified 400 times. ImageJ software was subsequently used to measure the diameters and cross-sectional areas of all muscle fibers in the HE-stained sections, along with the percentage of positively stained area in the Oil Red O-stained sections.

### Untargeted metabolomics profiling

#### Extraction of metabolites

First, 80 mg of the meat samples were mixed with 1 mL of precooled methanol: acetonitrile: water (2:2:1, vol/vol). Then, they were processed in liquid nitrogen, vortexed, sonicated, and centrifuged at 14,000 rpm for 20 min at 4°C. The supernatant was dried in a vacuum centrifuge and redissolved in 100 μL of mixture (acetonitrile: water = 1:1, vol/vol) for ultrahigh-performance liquid chromatography coupled with quadrupole time-of-flight mass spectrometry (UHPLC-QTOF-MS) analysis.

#### UHPLC-QTOF-MS analysis

A UHPLC system with a Hypesil Gold column (2.1 mm × 100 mm, 1.7 µm) coupled to an AB TOF 6600 mass spectrometer was used for subsequent analysis. The mobile phase system included solvent A (25 mM ammonium acetate and ammonium in water) and solvent B (acetonitrile). The gradient started at 85% B for 1 min, then decreased to 65% over 11 min, further decreased to 40% in 0.1 min and held for 4 min, then increased to 85% in 0.1 min and maintained for 5 min. Samples were stored in an autosampler at 5°C with a column temperature of 25°C, a flow rate of 0.4 mL/min, and an injection volume of 2 μL during analysis. Electrospray ionization (ESI)-positive and -negative ion modes were used for detecting metabolites. The ESI source conditions were set as follows: Ion Source Gas1, 60; Ion Source Gas2, 60; curtain gas (CUR), 30; source temperature, 600°C; IonSpray Voltage Floating (ISVF), ± 5,500 V; TOF MS instrument scan m/z range, 60–1,000 Da; product ion scan m/z range, 25–1,000 Da; TOF MS scan accumulation time, 0.20 s/spectra; and product ion scan accumulation time, 0.05 s/spectra. The product ion scan was acquired using information-dependent acquisition (IDA) in high-sensitivity mode. The parameters were set as follows: collision energy of 35 ± 15 eV, declustering potential (DP) of 60 V (positive mode) and −60 V (negative mode), excluding isotopes within 4 Da, and candidate ions to monitor per cycle of 10.

The raw MS data (wiff.scan files) for untargeted metabolomics were converted to MzXML files via ProteoWizard software. The XCMS program was used for peak alignment, detection, and retention time correction. Compound identification was performed by searching the laboratory’s custom-built database and public databases. Next, multivariate statistical analyses, including principal component analysis (PCA) and discriminant analysis of orthogonal partial least squares (OPLS-DA), were performed via SIMCA software v14.0. Heatmaps were created using the R package v3.4. The variable importance in the projection (VIP) of each variable in the OPLS-DA model was calculated to refine the analysis. The metabolites with VIP values exceeding one and the variables with *P* values< 0.05 were considered differentially abundant metabolites. The metabolites were annotated, and metabolic pathways were analyzed using the Kyoto Encyclopedia of Genes and Genomes (KEGG) database (https://www.genome.jp/kegg/). Pathways with *P* values< 0.05 were considered to be significant.

### Targeted metabolomics profiling of amino acids and their derivatives

The meat sample (50 mg) was combined with 500 µL of a mixture containing methanol, acetonitrile, and water at a 2:2:1 ratio. The sample was vortexed for 3 min and centrifuged at 12,000 rpm for 10 min at 4°C. The supernatant of the mixture was precipitated at –20°C for 30 min and centrifuged again at 12,000 rpm for 10 min at 4°C. After centrifugation, the supernatant was stored at –80°C for UPLC analysis. The mobile phase system included water with 2 mM ammonium acetate and 0.04% formic acid (A) and acetonitrile with 2 mM ammonium acetate and 0.04% formic acid (B). The samples were injected into an autosampler at 4°C, the column temperature was 40°C, the flow rate was 400 μL/min, and the injection volume was 2 μL. The gradient started at 90% B, decreased to 60% B at 9 min and 40% B at 10–11 min, and then returned to 90% B at 11–15 min. An AB 6500 QTRAP system was used to analyze the samples in both positive and negative ion modes. The ESI source was operated at a temperature of 550°C, with ion spray voltages of 5,500 V (positive) and –4,500 V (negative), and a curtain gas of 35. The multiple reaction monitoring (MRM) method was used for the quantitative acquisition of data via mass spectrometry. MultiQuant software was used for process alignment, peak detection, and retention-time corrections for amino acids and their derivatives for the targeted metabolomics data. The metabolites with *P* values< 0.05 were considered to be differentially abundant metabolites.

### 16S rDNA amplicon sequencing

Total genomic DNA from samples of intestinal contents was extracted using a Mag-binding soil DNA kit (Omega), and the purity and concentration of the DNA were tested. In accordance with the selection of the sequencing region, the selected V3–V4 variable region was amplified by PCR using specific primers with barcodes and high-fidelity DNA polymerase. The PCR products were detected via 2% agarose gel electrophoresis, and the target fragments were cut and recovered by a Quant iT PicoGreen dsDNA Assay Kit. In reference to the preliminary quantitative results of electrophoresis, the products recovered from the PCR amplification were detected and quantified with a microplate reader (BioTek, FLx800) fluorescence quantitative system, and the corresponding proportions were mixed according to the sequencing requirements of each sample. The library was constructed via the TruSeq Nano DNA LT Library Prep Kit from Illumina. The constructed library was inspected via an Agilent Bioanalyzer 2100 and a Promega QuantiFluor. After the library was qualified, it was sequenced.

The original sequencing data were preprocessed via Cutadapt software to detect and cut off the adapter. After trimming, the paired-end reads were filtered for low-quality sequences, denoised, merged, and detected, and the chimeric reads were removed via DADA2 with the default parameters of QIIME2. Finally, the software outputs the representative reads and the ASV abundance table. The representative read of each ASV was selected via the QIIME 2 package. All representative reads were annotated and blasted against the Silva database version 138 (16S rDNA) via classify-sklearn with the default parameters. The α- and β- diversity indices are calculated using QIIME 2 software. Alpha diversity is used to analyze the diversity of microbial communities within a sample, whereas beta diversity is a comparative analysis of the microbial community composition of different samples. For difference analysis, linear discriminant analysis effect size (LEfSe) was used to identify the species characteristics that explain the group differences.

### Data analysis

For univariate analysis, the differences in the phenotypic traits among the three energy groups were determined via one-way analysis of variance (ANOVA) via SPSS 10.0 software; significant differences were considered at *P* < 0.05. Pairwise Pearson’s correlation coefficients were generated to evaluate the correlations, and the correlation coefficients were visualized via heatmap plots via the R package “complex_heat_map”

## RESULTS

### Growth performance and carcass traits

When the chickens were 70 days old, no significant differences in body weight or frame size indicators were found among the three dietary treatments. By the end of the trial, live body weight showed a non-significant trend toward decreasing in the LE group and increasing in the HE group relative to the ME control (*P* > 0.05). However, the HE group exhibited a significantly greater interpubic width than both the ME and LE groups (*P* < 0.05) (Table 2). Post-slaughter measurements revealed a similar trend. While carcass weight in the LE group decreased and that in the HE group increased relative to the ME control, neither difference reached statistical significance (*P* > 0.05). The liver weight in the HE group was significantly greater than that in the control ME group (*P* < 0.05). No significant differences in carcass yield or pectoral muscle, leg muscle, abdominal fat, oviduct, gizzard, or glandular stomach weights were found among the treatment groups (*P* > 0.05). Different dietary energy levels significantly affected the intestinal coefficient and length of the duodenum and jejunum but not the ileum (*P* < 0.05) (Table 3).

**TABLE 2:** The effect of different dietary energy levels on body weight and frame size of chicken^*a*^^,*b*^

Items(cm)	Traits				
	LE	ME	HE	SEM	*P*-value
70 days					
Live body weight	601.70	646.90	618.90	8.90	0.11
Body slope length	15.00	15.94	16.02	0.21	0.09
Sternal length	8.20	8.64	8.26	0.16	0.51
Tibia length	85.58	87.16	87.64	1.01	0.71
Tibial circumference	3.42	3.40	3.46	0.04	0.82
Interpubic width	0.94	0.93	0.96	0.11	0.53
140 days					
Live body weight	1,436.60^B^	1,496.70^AB^	1,588.70^A^	22.47	0.01
Body slope length	18.96	20.82	18.78	0.44	0.12
Sternal length	15.15	15.56	15.91	0.22	0.39
Tibia length	98.54	102.41	99.16	1.09	0.32
Tibial circumference	4.53	4.60	4.79	0.05	0.06
Interpubic width	1.63^B^	1.71^B^	2.02^A^	0.06	0.02

^*a*^ LE, low energy; ME, medium energy; HE, high energy；SEM, standard error of the mean.

^*b*^ Values within a row with different superscript letters (A and B) differ significantly (*P* < 0.05).

**TABLE 3:** The effect of different dietary energy levels on carcass traits of chicken^*a*^^,^^*b*^

Items	Traits				
	**LE**	**ME**	**HE**	**SEM**	***P*-value**
Carcass weight (g)	1,113.40^B^	1,149.00^AB^	1,236.10^A^	31.86	0.03
Carcass yield (%)	77.51	76.76	77.84	0.73	0.83
Liver weight (g)	23.96^B^	24.38^B^	35.17^A^	1.62	0.002
Pectoral muscle weight (g)	130.86	127.38	140.81	2.95	0.16
Leg muscle weight (g)	158.55	159.50	167.73	2.25	0.24
Abdominal fat weight (g)	28.09	36.91	37.95	4.14	0.60
Oviduct weight (g)	35.32	39.45	42.74	1.84	0.11
Gizzard weight (g)	24.63	23.16	27.91	1.19	0.27
Glandular stomach weight (g)	5.28	5.05	5.72	0.19	0.36
Intestinal coefficient (%)					
Duodenum	0.84^A^	0.65^B^	0.71^B^	0.05	0.04
Jejunum	1.77^A^	1.15^B^	1.17^B^	0.2	＜0.001
Ileum	1.41	1.16	1.07	0.1	0.19
Intestinal length (cm)					
Duodenum	20.40^B^	21.40^B^	24.60^A^	0.59	0.006
Jejunum	52.00^A^	52.55^A^	44.60^B^	1.17	0.005
Ileum	48.20	50.10	46.90	1.25	0.60

^*a*^ LE, low energy; ME, medium energy; HE, high energy.

^*b*^ Values within a row with different superscript letters (A and B) differ significantly (*P* < 0.05).

### Meat phenotype characteristics

The effects of dietary energy level on the meat quality of chickens are presented in Table 4. The edible quality of the meat was indicated by pH_24h_, drip loss, cooking loss, and color. Although the values of pH_24h_, cooking loss, redness, and yellowness were greater in the HE group than in the ME and LE groups, no significant differences were detected among the three groups (*P* > 0.05). Compared with the control (ME) group, the HE diet resulted in a significant increase in drip loss (*P* < 0.05). The lightness was the highest in the ME group.

**TABLE 4:** The effect of different dietary energy levels on meat quality of chicken^*a*^^,^^*b*^

Items		Traits				
		LE	ME	HE	SEM	*P*-value
pH_24h_		5.59	5.61	5.61	0.01	0.71
Drip loss (%)		2.53^B^	2.33^B^	3.87^A^	0.25	0.022
Cooking loss (%)		19.82	20.10	22.16	0.56	0.18
Color	Lightness(L^*^)	40.65^B^	42.61^A^	40.31^B^	1.19	0.031
	Redness(a^*^)	3.28	3.41	3.45	0.44	0.90
	Yellowness(b^*^)	9.81	10.19	10.15	0.83	0.82
Moisture		70.50	70.40	70.20	0.20	0.83
IMF		4.21^B^	4.86^A^	5.21^A^	0.17	<0.01
Protein		22.86^B^	24.43^A^	24.98^A^	0.57	<0.01
IMP		2.09	2.10	2.10	0.01	0.96

^*a*^ LE, low energy; ME, medium energy; HE, high energy.

^*b*^ Values within a row with different superscript letters (A and B) differ significantly (*P* < 0.05).

**Fig 2:**
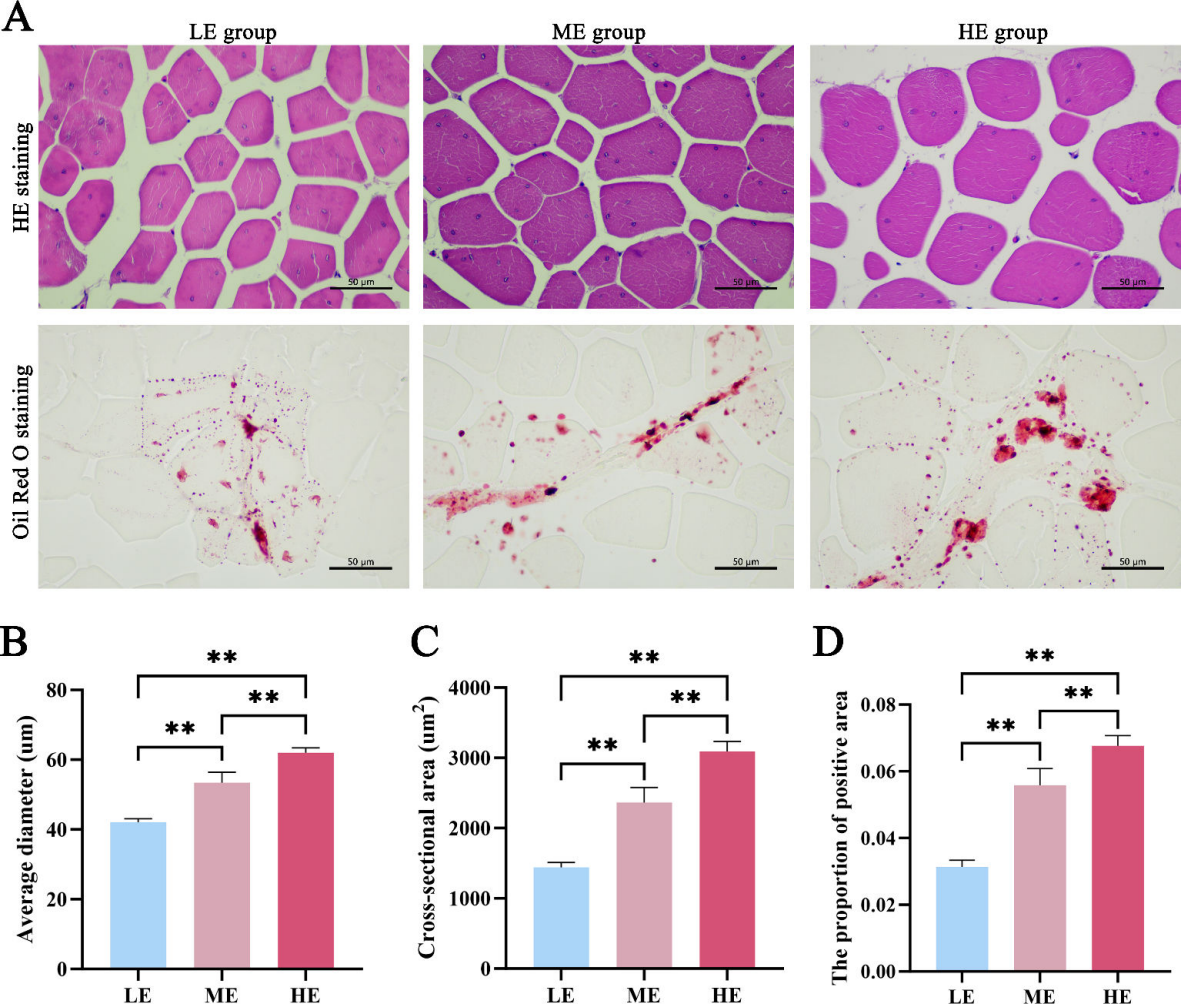
(**A**) HE staining and Oil Red O-stained section of muscle fibers in samples with different energy levels of diets (400×). (**B**) Average diameter of muscle fibers in different groups. (**C**) Cross-sectional area of muscle fibers in different groups. (**D**) Percentage of positive area in Oil Red O-stained section. * *P* < 0.05, ** *P* < 0.01.

The conventional nutrients of meat are mainly reflected by indicators such as moisture, protein, and fat contents. There was no significant effect on moisture or IMP content (*P* > 0.05). Compared to the ME control, the LE diet significantly reduced both IMF and protein content (*P* < 0.01). Morphometric analysis revealed that both the average diameter and cross-sectional area of muscle fibers increased with increasing dietary energy levels (*P* < 0.01) (Fig. 2). Consistent with the chemical analysis of IMF, Oil Red O staining indicated that the area of lipid deposition within the muscle was greatest in the HE group, followed by the ME and LE groups, visually confirming the graded effect of dietary energy on intramuscular fat accumulation.

### Untargeted metabolomics

#### Differential metabolite identification

**Fig 3:**
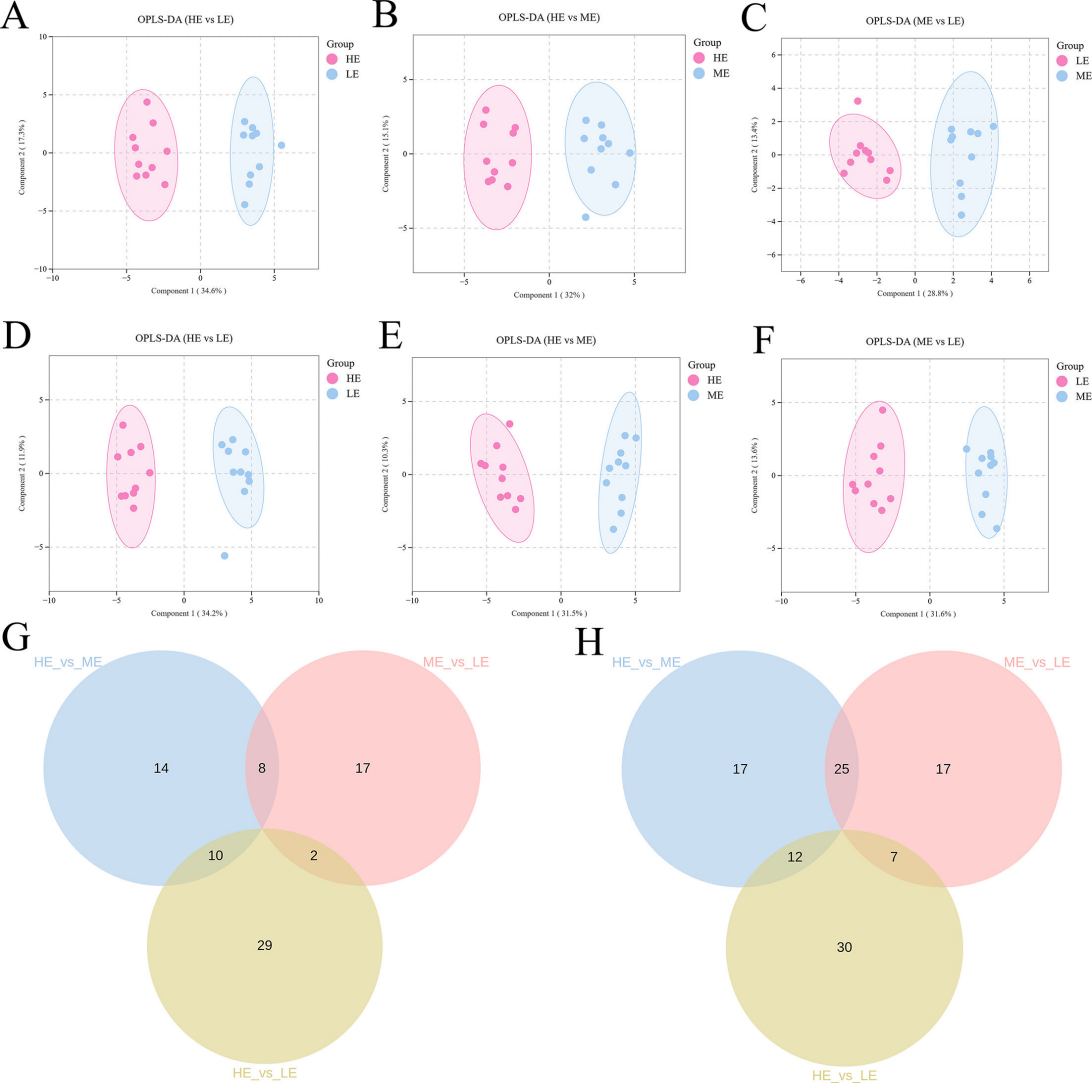
OPLS-DA scores of the overall sample in the positive ion detection mode (**A**) HE vs. LE, (**B**) HE vs. ME, and (**C**) ME vs. LE. OPLS-DA scores of the overall sample in the negative ion detection mode (**D**) HE vs. LE, (**E**) HE vs. ME, and (**F**) ME vs. LE. Venn diagram of three pairwise comparisons (HE vs. LE, HE vs. ME, and ME vs. LE) in the positive ion detection mode (**G**) and negative ion detection mode (**H**).

In the positive ion detection mode, we performed OPLS-DA to characterize the similarities and differences among all the samples in the three groups (Fig. 3A through C). The developed model demonstrated stability and reliability and was able to effectively compare the differences between the two groups (Fig. S1A through D). On the basis of the cutoff (VIP > 1 and *P* < 0.05), a total of 80 differentially abundant metabolites (DMs) were detected, with 32 between the HE and ME groups, 27 between the ME and LE groups, and 41 between the HE and LE groups.

In the negative ion detection mode, OPLS-DA also revealed metabolite segregation among all the samples in the three groups (Fig. 3D through F). The model validation of OPLS-DA was shown in Fig. S1E through H. Based on the same screening conditions, a total of 108 DMs were identified (54 between the HE and ME groups, 49 between the ME and LE groups, and 49 between the HE and LE groups). In both modes, the Venn diagram revealed no commonly differential molecules in the three pairwise comparisons (Fig. 3G and 2H).

**Fig 4:**
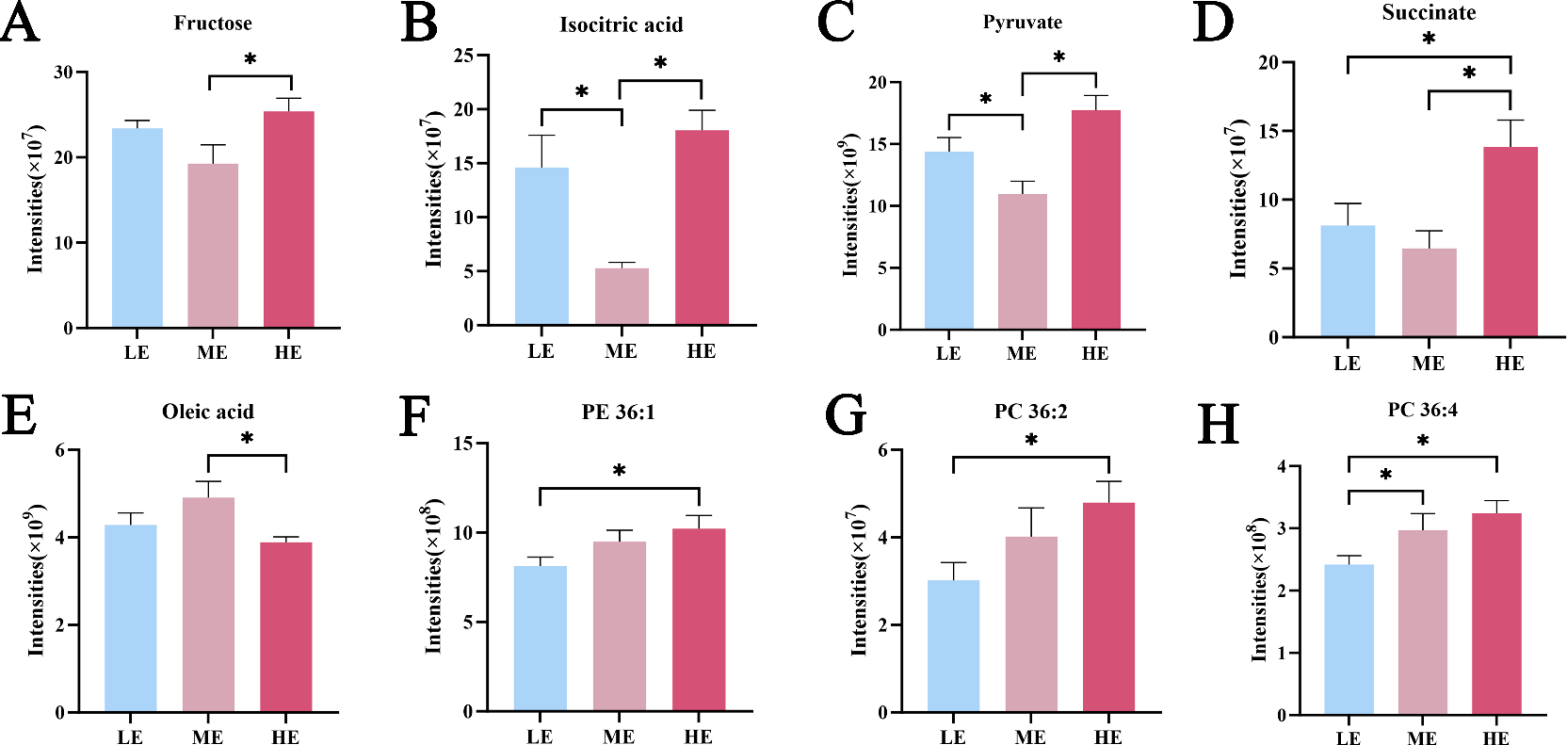
The relative peak intensities of key metabolites associated with energy metabolism (**A–D**) and lipid metabolism (**E–H**). *P < 0.05.

The influence of different dietary energy levels on amino acid metabolites was quite prominent, with 39 amino acid metabolites identified. Key intermediates of central energy metabolism were significantly altered. Compared to the ME control group, the abundances of fructose, isocitric acid, pyruvate, and succinate were significantly greater in the HE group (*P* < 0.05) (Fig. 4A through D). Notably, the levels of isocitric acid and pyruvate were also significantly elevated in the LE group relative to the ME control (*P* < 0.05). The contents of specific glycerophospholipids (PC 36:2, PC 36:4, and PE 36:1), which are associated with meat flavor and nutritional quality, were not significantly different between the HE and ME groups. However, the HE group exhibited significantly higher levels of these phospholipids than the LE group (*P* < 0.05) (Fig. 4F through H). Conversely, the monounsaturated fatty acid oleic acid was most abundant in the ME control group, showing significantly higher levels than in the HE group (*P* < 0.05) (Fig. 4E).

#### Differentially abundant metabolite pathway analysis

**Fig 5:**
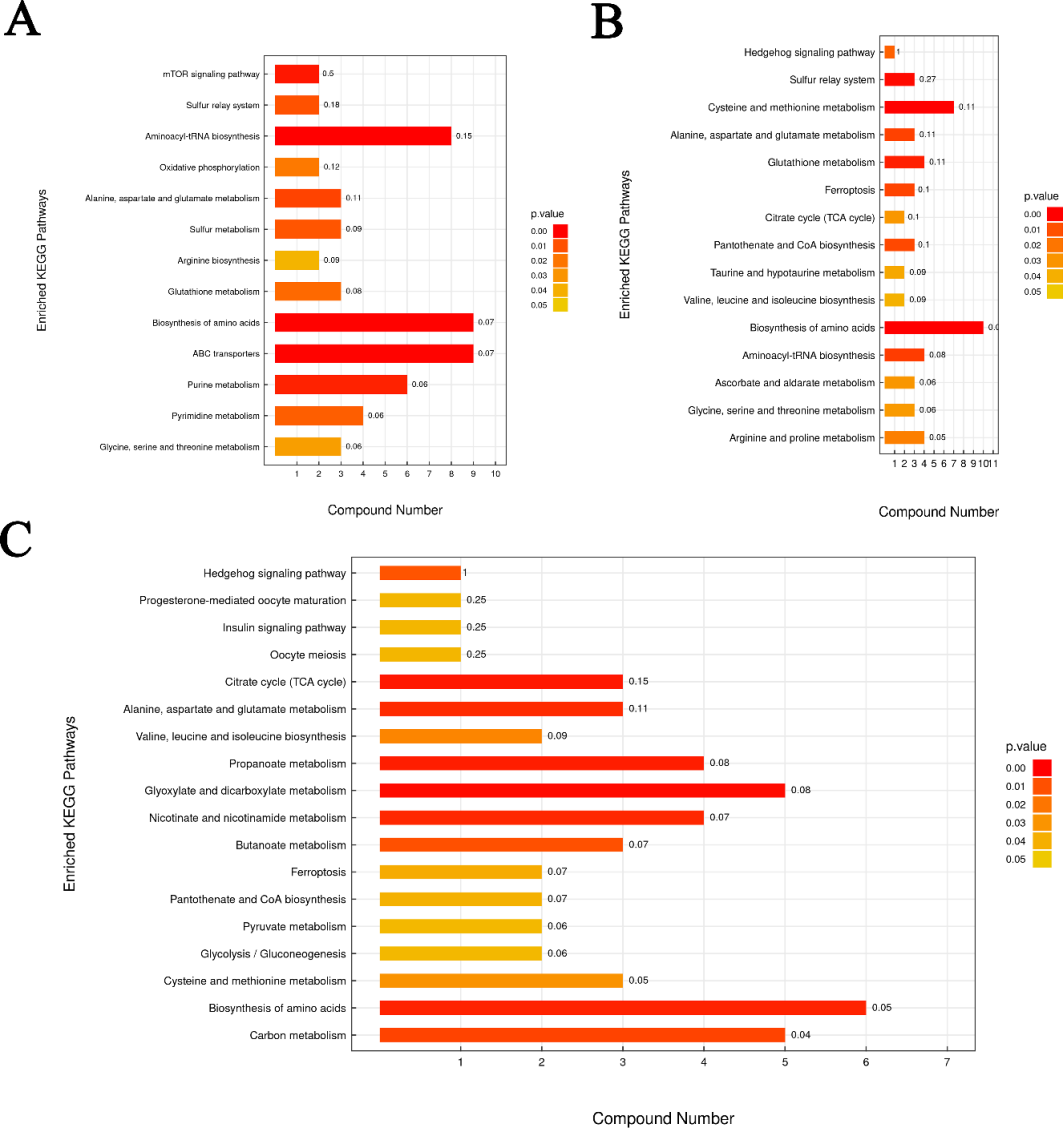
The enrichment of KEGG metabolic pathways of differential metabolites in the breast muscle of chicken in the HE vs. LE (**A**), HE vs. ME (**B**), and ME vs. LE (**C**) comparisons.

We used KEGG to further analyze the functions of the differentially abundant metabolite-related pathways. The metabolites identified in both positive and negative ion modes were combined to enhance comprehensiveness and support credibility. The results revealed 13, 15, and 18 significantly enriched metabolic pathways in the HE vs. LE, HE vs. ME, and ME vs. LE comparisons, respectively (*P* < 0.05). There were two common pathways in the three comparisons, namely, biosynthesis of amino acids and alanine, aspartate, and glutamate metabolism. The differentially abundant metabolites enriched in these pathways were DL-asparagine, glycine, arginine, D-glutamine, DL-cysteine, L-methionine, leucine, O-acetyl-l-serine, phenylalanine, alpha-ketoisovaleric acid, isocitric acid, pyruvate, S-adenosyl-l-homocysteine, S-adenosyl-methionine, dihydroxyacetone phosphate, glutamine, and succinate. Moreover, energy metabolism-related pathways were identified in the ME vs. LE comparison (*P* < 0.05). Isocitric acid, pyruvate, and succinate were enriched in the citrate cycle and glyoxylate cycle (Fig. 5).

### Targeted metabolomic analysis

**Fig 6:**
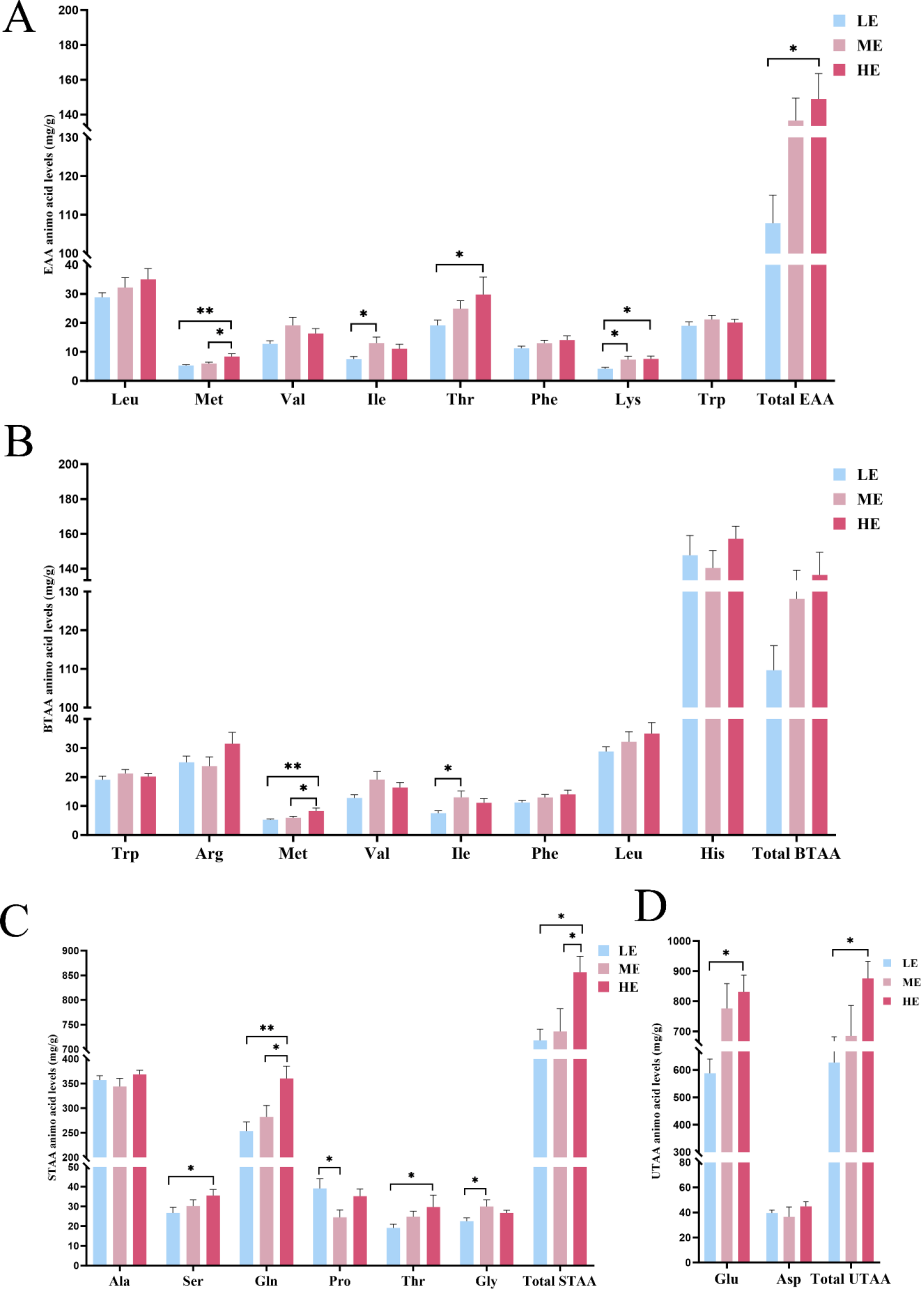
(**A**) The content of individual and total EAA among three dietary energy groups (Total EAA = Leu + Met + Val + Ile + Thr + Phe + Lys + Trp). (**B**) The content of individual and total BTAA among three dietary energy groups (Total BTAA = Trp + Arg + Met + Val + Ile + Phe + Leu + His). (**C**) The content of individual and total STAA among three dietary energy groups (Total STAA = Ala + Ser + Gln + Pro + Thr + Gly). (**D**) The content of individual and total UTAA among three dietary energy groups (Total UTAA = Glu + Asp). * *P* < 0.05, ** *P* < 0.01.

The results of untargeted metabolomics revealed that the effects of different dietary energy levels on amino acid metabolites were quite prominent. Thus, we conducted a targeted metabolomics study of amino acids to confirm the above speculation regarding the deposition of amino acids. In total, 68 amino acid metabolites were detected in all the samples. Anserine was the most abundant amino acid in chicken meat, with average values of 8,748.04, 8,497.07, and 8,548.99 mg/g in the LE, ME, and HE groups, respectively. A general trend of increasing total essential amino acid (EAA) content was observed with elevated dietary energy. While the HE group showed significantly greater total EAA content than the LE group (*P* < 0.05), neither the HE nor the LE group differed significantly from the ME control. The main EAAs that differed among the three groups included L-lysine, L-methionine, and L-threonine, thus contributing to the differences in the EAA content (Fig. 6A). Different amino acids contribute to different taste categories. Other studies that analyzed different tastes of amino acids have divided the amino acids into bitter-taste amino acids (BTAAs), sweet-taste amino acids (STAAs), and umami-taste amino acids (UTAAs) (18–20). The most abundant BTAA in chicken meat was L-histidine. Comparisons against the ME control revealed significant changes in specific BTAAs: L-methionine was higher in the HE group, while L-isoleucine was lower in the LE group (*P* < 0.05). Despite these individual differences, the total BTAA content remained similar among the three dietary groups (*P* > 0.05) (Fig. 6B). The dietary energy level significantly affected the content of STAA. L-alanine and L-glutamine were found to be the dominant STAAs in chicken meat (Fig. 6C). While L-serine and L-threonine levels were significantly higher in the HE group than in the LE group (*P* < 0.05), they did not differ from the ME control. Notably, the LE diet altered specific STAAs differently from the control, increasing L-proline but decreasing glycine compared to the ME group (*P* < 0.05). Similar to the pattern found in the EAA results, the HE group presented the highest content of total STAA, followed by the ME group and the LE group (*P* < 0.05) (Fig. 6C). For UTAAs, L-glutamic acid was the most abundant representative and the major free amino acid. While L-glutamic acid and total UTAA contents were significantly higher in the HE group than in the LE group (*P* < 0.05), they did not differ significantly from levels in the ME control group (Fig. 6D). These findings support the increase in downstream metabolites observed in the biosynthesis of amino acids during untargeted analysis.

### Cecal microbial community composition

**Fig 7:**
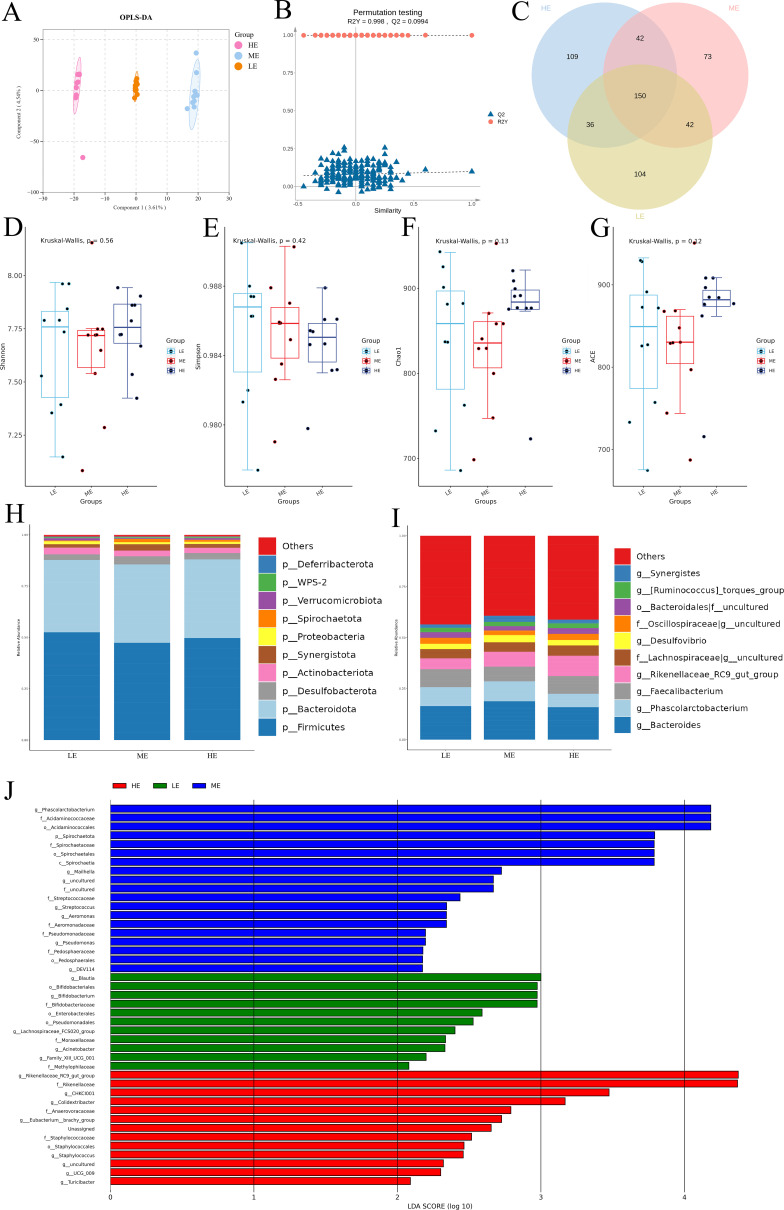
(**A**) OPLS-DA taxonomical classifications of cecum bacteria communities among three dietary energy groups. (**B**) The permutation test of OPLS-DA. (**C**) Venn diagram illustrating the overlaps of microbial ASVs among three dietary energy groups. (**D–G**) Alpha diversity of the cecum bacteria among three dietary energy groups. (**H**) Relative abundance of was bacteria community proportions at the phylum level. (**I**) Relative abundance of bacteria community proportions at the genus level. (**J**) Histogram of the linear discriminant analysis (LDA) effect among the three groups, and the LDA score (log10) > 2 was shown.

The OPLS-DA plot revealed that there was a significant obvious separation among the three groups (Fig. 7A). The model validation of OPLS-DA was shown in Fig. 7B. The Venn diagram shows that a total of 556 differential microorganisms were detected, with 150 differential microorganisms among three dietary energy groups (Fig. 7C).

The addition of elevated dietary energy had no significant effect on the Shannon, Simpson, Chao1, or ACE indices of bacterial richness and diversity (*P* > 0.05) (Fig. 7D through G). At the phylum level, the cecal microbiota of the three groups was mainly composed of Bacteroidetes and Firmicutes (Fig. 7H). The abundance of Firmicutes was significantly greater in the LE group than in the ME control (*P* < 0.05). At the genus level, specific taxa responded differentially to energy intake (Fig. 7I). Compared to the ME control, the HE group exhibited a significant decrease in the abundance of *Phascolarctobacterium* (*P* < 0.05). In contrast, the *Rikenellaceae_RC9_gut_group* in the HE group was significantly greater than that in the other two groups (*P* < 0.05). The ME control group was distinguished by a significantly higher abundance of *Synergistes* compared to both the HE and LE groups (*P* < 0.05). The *Oscillospiraceae uncultured* was obviously enriched in the HE group and LE group relative to the ME control (*P* < 0.05).

The linear discriminant analysis (LDA) effect size results revealed that 43 genera were markers that distinguished the three groups of samples (Fig. 7J). Eleven genera were enriched in the LE group: *o__Bifidobacteriales, o__Enterobacterales, o__Pseudomonadales, f__Bifidobacteriaceae, f__Moraxellaceae, f__Methylophilaceae, g__Bifidobacterium, g__Acinetobacter, g__Blautia, g__Lachnospiraceae_FCS020_group* and *g__Family_XIII_UCG_001*. Nineteen genera were significantly associated with the ME group: *p__Spirochaetota, c__Spirochaetia, o__Acidaminococcales, o__Spirochaetales, o__Pedosphaerales, f__Acidaminococcaceae, f__Spirochaetaceae, f__Streptococcaceae, f__uncultured, f__Aeromonadaceae, f__Pseudomonadaceae, f__Pedosphaeraceae, g__Phascolarctobacterium, g__Mailhella, g__Streptococcus, g__uncultured, g__Aeromonas, g__Pseudomonas* and *g__DEV114*. Thirteen genera were strongly enriched in the HE group: Unassigned, *o__Staphylococcales, f__Rikenellaceae, f__Anaerovoracaceae, g__Rikenellaceae_RC9_gut_group, g__CHKCI001,  g__Colidextribacter, g___Eubacterium__brachy_group, g__UCG_009,  g__uncultured,  g__Staphylococcus, g__Turicibacter,* and *f__Staphylococcaceae*.

### Correlation analysis

**Fig 8:**
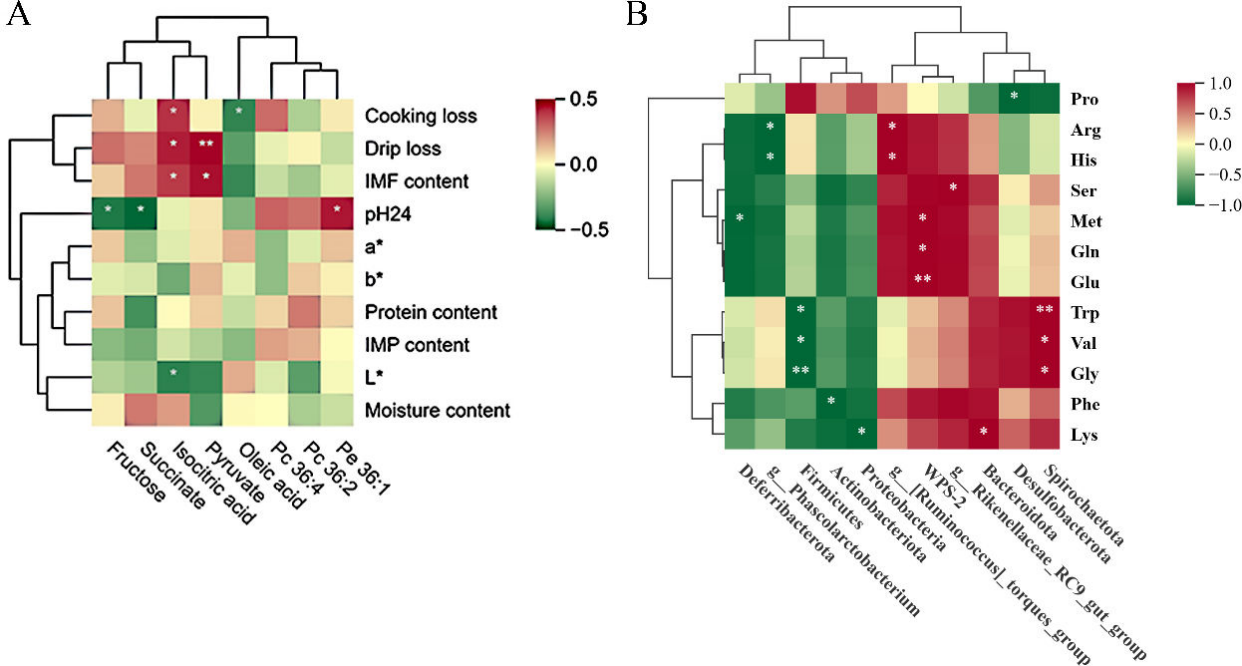
(**A**) The correlation heat map between meat quality characters and important metabolites. (**B**) The correlation heat map between cecal microbial and amino acids. The red color and green color represent positive and negative correlations, respectively. * *P* < 0.05, ** *P* < 0.01.

The correlations between the muscle metabolites and meat quality parameters in chickens fed a diet with different energy levels were evaluated via metabolomics and phenotypic data (Fig. 8A). The results revealed that the levels of isocitric acid and pyruvate were positively correlated with the IMF content (*P* < 0.05). Moreover, isocitric acid was positively correlated with drip loss and cooking loss (*P* < 0.05), and pyruvate was highly significantly positively correlated with drip loss (*P* < 0.01). The value of pH_24h_ was negatively correlated with fructose and succinate, and a negative correlation was found between the L value and isocitric acid content (*P* < 0.05).

The relationships between the cecal microbiota and muscle amino acid profiles are summarized in Fig. 8B. Across the spectrum of dietary energy levels, correlation analysis revealed several significant associations. Correlation analysis revealed that L-tryptophan, glycine, L-lysine, and L-valine were significantly negatively correlated with the abundances of Firmicutes and Proteobacteria, whereas they were significantly positively correlated with the abundances of *Spirochaetota* and *Bacteroides* (*P* < 0.05). L-methionine, L-glutamic acid, and L-glutamine contents were positively correlated with the abundance of WPS-2 (*P* < 0.05). L-proline was negatively correlated with *Desulfobacterota* (*P* < 0.05). There was a negative correlation between L-phenylalanine and the abundance of Actinobacteria (*P* < 0.05). L-methionine was negatively correlated with *Deferibacterota* (*P* < 0.05). These correlative patterns provided a potential microbial-mediated mechanism underlying the observed variations in meat quality among chickens fed different energy diets.

## DISCUSSION

In the poultry industry, providing adequate nutrients is the most important factor for achieving efficient production. In this study, the increase in dietary energy improved the live body weight, interpubic width, carcass weight, and liver weight, whereas the increase in dietary energy level did not influence other body frame sizes or other carcass components. A similar finding that low energy levels reduced body weight was also reported in another study (21). Holsheimer et al. reported that high-energy and high-protein diets increased the yield of the breast and the weight of the carcass (22). In previous studies on rapidly growing broilers, different energy levels in the diet did not influence broiler weight or carcass weight (23, 24). Due to differences in chicken breeds, these studies used a relatively high energy concentration gradient, and the trial lasted 42 days. Rosa et al. used diets with 2,950, 3,200, and 3,400 kcal/kg metabolizable energy and reported no effect on carcass yield, although an increase in energy concentration increased the abdominal fat content (25).

Meat quality is an important economic trait in chickens (26). The nutrient composition of the diet is the most important factor in determining raw meat quality (27–29). Understanding how energy is utilized by chickens and determining the optimal dietary energy level for the best quality chicken meat is essential in the poultry industry. In a study on chickens subjected to six dietary metabolizable energy treatments, the energy concentration did not affect the breast muscle pH (at 45 min and 24 h), color parameter (L*), or percentage of drip loss (*P* > 0.05) (30). Water holding capacity is one of the most important traits of meat quality. The losses when cut (called drip loss) or cooked (called cooking loss) directly translate to financial loss when delivering meat to the customer (31). In this study, cooking loss was greater for chicken meat that was fed high-energy diets. An increased dietary energy level possibly accelerated more glycogen phosphorylase denaturation to shift the protein from the sarcoplasmic to the myofibrillar protein fraction, thus resulting in a lower water-holding capacity (32).

The protein content is an important indicator of the nutritional value of breast meat (33), while IMF is also an important indicator of flavor, juiciness, and palatability (34). Liu et al. reported that the pectoral IMF content of rapidly growing broilers was lower after being fed diets with lower energy levels (35). The pectoral IMF content increased gradually with increasing dietary energy levels in Beijing-You chickens, which are also native chicken breeds (4). Compared with the recommended energy level (ME), increased dietary energy levels did not significantly affect the IMF content in our study. To limit excess energy loss from the diet, a medium energy level was sufficient to yield meat with similar IMF contents.

The quality of meat is strongly influenced by muscle metabolites. Metabolomics is an emerging technique for identifying and characterizing small molecules. Using the UHPLC-QTOF-MS-based metabolomics approach, we found that glycerophospholipid metabolites (PC 36:2, PC 36:4, and PE 36:1) presented the highest abundance in the HE group. The phospholipids in intramuscular fat are closely related to meat flavor and nutritional value (34, 36). In terms of glycerophospholipid abundance, the HE groups may have better meat flavor. Oleic acid is an omega-9 monounsaturated fatty acid that can reduce blood pressure and prevent type 2 diabetes mellitus and cardiovascular diseases (37). A significantly greater intensity of oleic acid was found in the ME group. Additionally, substances implicated in energy metabolism, including fructose, isocitric acid, pyruvate, and succinate, were the most abundant in the HE group. Pyruvate, the terminal product of glycolysis, plays crucial roles in aerobic and anaerobic oxidation, as well as in the synthesis and oxidation of lipids (36). Fructose carbon enters glycolysis via fructose-1-phosphate. Fructose intake leads to obesity and hyperlipidemia (38). On the basis of these findings, the lowest intensity of fructose in the ME group may be healthier than that in the other two groups.

The quality of flavor, which serves as a crucial parameter in the sensory assessment of meat quality, is a significant attribute that influences the choices of consumers (20). Free amino acids constitute one of the major flavor and aroma precursors in chicken meat. Many amino acids have sweet, sour, salty, fresh, and other flavors and can be roughly categorized into STAA, BTAA, and UTAA. These higher levels of Ser, Gln, and Thr in the breast muscle of the HE group relative to those in the LE and ME groups increased the sweetness of the meat. Aspartate and glutamic acid, known as umami amino acids, are important umami substances in meat (19, 39). The content of L-glutamic acid was the highest in chickens fed high-energy diets. Muscular pipecolic acid is used to synthesize glutamate and is an important intermediate in the metabolism of Lys (40). The HE group presented the highest contents of glutamate and Lys but the lowest content of pipecolic acid, which reflects the process by which pipecolic acid is consumed to synthesize glutamate and Lys. EAAs serve as the foundational components of proteins crucial for sustaining physiological processes in humans; they must be obtained from foods because they cannot be biosynthesized in the body (18). In this study, we suggested that an increase in dietary energy level could significantly increase the EAA content, which is in agreement with the findings of Chang et al. Thus, a dietary energy level of 13.06 MJ/kg, which is greater than the nutritional requirements recommended for Chinese local chicken breeds, has beneficial effects on improving meat quality in terms of amino acid composition (41).

Our findings elucidate the distinct trade-offs between high- (HE) and low-energy (LE) diets relative to the medium-energy control (ME), with clear implications for both consumers and the poultry industry. For consumers, the HE diet offered superior nutritional value (highest EAA) and enhanced umami and sweet-taste potential linked to specific amino acids, but at the cost of significantly higher drip loss, which reduced edible yield and juiciness. Conversely, the LE diet yields leaner meat with minimal processing loss; however, it suffers from substantially lower protein and EAAs content, compromising its nutritional value. The ME diet provided a balanced compromise, delivering reliable quality without these extremes. From an industry perspective, the HE formulation, which included elevated levels of soybean oil and maize gluten meal, likely incurred a higher ingredient cost per unit compared to the ME diet, in which wheat bran served as a lower-cost filler (42). The LE diet minimizes feed cost but extends the production cycle due to slower growth, while the ME diet represents the most cost-effective and low-risk strategy for mainstream production, optimizing the balance between input cost, growth efficiency, and final product quality. Thus, the choice of dietary energy strategy should be market-driven: HE for premium quality segments, LE for lean or cost-driven production, and ME for efficient conventional production.

The differential enrichment of specific cecal microbiota genera across the dietary groups provides a plausible biological link between diet and meat quality. The enrichment of *Rikenellaceae_RC9_gut_group* in the HE group is particularly notable, as members of this genus are often associated with efficient fiber fermentation and the production of short-chain fatty acids (SCFAs) like acetate and propionate (43). These SCFAs can serve as energy substrates or signaling molecules that may influence host metabolism (44). This could partly explain the enhanced amino acid deposition observed in the HE group. In contrast, the ME group’s unique enrichment of *Synergistes* suggested a microbiome adapted to a balanced diet, potentially supporting stable nutrient absorption (45). The LE group’s higher abundance of Firmicutes, a phylum often linked to energy harvest, might reflect a compensatory mechanism to extract maximal energy from a nutrient-dilute diet, albeit insufficient to support optimal muscle accretion (46). These distinct microbial consortia likely contribute to the differential metabolic and nutritional outcomes observed in the muscle.

Importantly, the gut microbiota plays a pivotal role in the digestion and metabolism of amino acids, which are the building blocks of protein and are essential for muscle development and meat quality in animals (13, 47). The results of the present study indicated that the contents of *Spirochaetota, Bacteroides,* and *Rikenellaceae_RC9_gut_group* bacteria in the cecum microflora increased with increasing dietary energy. This increase had an impact on the amino acid concentration of the muscle and, consequently, on the meat quality. The findings of Li et al. also suggest that a balanced gut microbiota could increase the bioavailability of amino acids, thereby supporting muscle growth and potentially improving the nutritional value of meat (48). Lactobacillus, a probiotic bacterium, enhances the bioavailability of essential amino acids, as demonstrated by Sumi et al., potentially improving muscle protein accretion (49). Moreover, the gut microbiota can influence meat quality through its impact on fatty acid metabolism. For example, butyric acid, produced by certain gut bacteria, has been linked to increased intramuscular fat deposition, which can affect the tenderness and flavor of meat (50). Thus, modifying the gut microbiota through dietary intervention represents a promising strategy for improving meat quality in poultry production.

Finally, it is important to acknowledge the limitations of this study. While strong correlations were established between the cecal microbiota and amino acids in muscles, the study design does not confirm causality. Future research employing techniques like fecal microbiota transplantation is needed to definitively prove that the observed microbial shifts directly cause the changes in muscle amino acid composition. Furthermore, the economic analysis presented is preliminary. A comprehensive assessment, factoring in total feed cost, growth rate, feed conversion ratio, processing yield, and potential market price premiums, is required to provide definitive economic guidance to producers. Addressing these limitations in future work will strengthen the practical application of these findings.

### Conclusion

In summary, this comparative study demonstrates that low, medium, and high dietary energy levels distinctly resulted in different phenotypic and metabolic profiles in local chickens. The HE diet promoted superior growth and enriched muscle amino acid profiles; however, it concurrently increased drip loss and raised feed costs. In contrast, the LE diet was the most economical and yielded the leanest meat, though this came at the expense of reduced protein and key amino acids. The ME control diet offered an efficient balance, supporting steady growth while maintaining reliable meat quality and a favorable oleic acid level. Integrated multi-omics analysis revealed that dietary energy level potentially influenced muscle metabolism and amino acid composition by remodeling the cecal microbial community. Together, these findings provide a scientific framework for selecting dietary energy strategies tailored to specific market demands: HE for premium quality segments, LE for lean or cost-driven production, and ME for efficient conventional production.

## Data Availability

Upon reasonable request, the data sets of this study can be made available from the corresponding author.

## SUPPLEMENTAL MATERIAL

Supplemental material spectrum.02046-25-s0001.docx Table S1; Fig. S1.

ASM does not own the copyrights to Supplemental Material that may be linked to, or accessed through, an article. The authors have granted ASM a non-exclusive, world-wide license to publish the Supplemental Material files. Please contact the corresponding author directly for reuse.
