# Supplementary material for: Dietary energy level modulates cecal microbiota to influence amino acid composition of local chicken meat
Source: Microbiol Spectr. 2026 Mar 23;14(5):e02046-25. doi: 10.1128/spectrum.02046-25 (PMC13141916; doi:10.1128/spectrum.02046-25)
Supplement: Supplemental material — Table S1; Fig. S1. [file spectrum.02046-25-s0001.docx]

Table S1. Differential metabolites discriminating chicken meat from the other two groups

| Compound_ID | Compounds | m/z | RT(s) | Superclass | Compared with the other two groups |
| --- | --- | --- | --- | --- | --- |
| **Differential metabolites in LE group** | | | | | |
| M311T27 | Thymol-beta-d-glucoside | 413.08 | 27.29 | Lipids and lipid-like molecules | up |
| M333T413 | Diflufenzopyr | 333.06 | 412.95 | Benzenoids | up |
| M329T37 | (z)-9,12,13-trihydroxyoctadec-15-enoic acid | 329.25 | 36.73 | Lipids and lipid-like molecules | up |
| M297T28 | Ostruthin | 297.15 | 27.87 | Lipids and lipid-like molecules | up |
| M327T45 | Cis-4,7,10,13,16,19-docosahexaenoic acid | 327.23 | 45.05 | Lipids and lipid-like molecules | up |
| M841T136 | Pc 36:4 | 840.58 | 135.66 | Lipids and lipid-like molecules | up |
| M135T35 | 3,4-dimethylbenzaldehyde | 135.08 | 34.87 | Benzenoids | down |
| M117T366 | Succinate | 117.02 | 365.83 | Organic acids and derivatives | down |
| M167T116 | Cyromazine | 167.10 | 116.18 | Organoheterocyclic compounds | down |
| **Differential metabolites in ME group** | | | | | |
| M397T26_2 | Mitragynine | 397.23 | 26.34 | Undefined | up |
| M497T27 | Poricoic acid a | 497.32 | 26.61 | Lipids and lipid-like molecules | up |
| M399T421 | S-Adenosylmethionine | 399.14 | 421.25 | Nucleosides, nucleotides, and analogues | up |
| M381T25 | 5-heptenoic acid | 381.23 | 25.15 | Lipids and lipid-like molecules | up |
| M425T26 | 2,6,10-trienoic acid | 425.26 | 25.97 | Lipids and lipid-like molecules | up |
| M453T26 | 3-ketofusidic acid | 453.29 | 25.63 | Lipids and lipid-like molecules | up |
| M485T28 | Kendomycin | 485.28 | 28.28 | Undefined | up |
| M281T27 | Octyl gallate | 281.14 | 26.64 | Benzenoids | up |
| M529T29 | Kukoamine a | 529.31 | 29.45 | Benzenoids | up |
| M288T192 | Octanoylcarnitine | 288.22 | 192.38 | Lipids and lipid-like molecules | up |
| M341T31 | 9-phenyl-1-(2,4,6-trihydroxyphenyl)nonan-1-one | 341.18 | 30.72 | Organic oxygen compounds | up |
| M103T231 | 3-hydroxybutyric acid | 103.04 | 231.29 | Organic acids and derivatives | up |
| M119T237 | Purine | 119.04 | 236.83 | Organoheterocyclic compounds | up |
| M441T27_1 | 6,8-dihydroxy-2,2,4,4-tetramethyl-7-(3-methylbutanoyl)-9-(2-methylpropyl)-9h-xanthene-1,3-dione | 441.25 | 27.29 | Organoheterocyclic compounds | up |
| M103T183 | Dl-a-hydroxybutyric acid | 103.04 | 183.22 | Organic acids and derivatives | up |
| M353T25_2 | Rauwolscine | 353.20 | 25.49 | Undefined | up |
| M306T380_2 | L-glutathione, reduced | 306.08 | 380.14 | Organic acids and derivatives | down |
| M413T291 | .alpha.-d-glucose pentaacetate | 413.08 | 291.33 | Organic acids and derivatives | down |
| M114T296 | D-proline | 114.06 | 295.81 | Organic acids and derivatives | down |
| M143T319 | Ectoine | 143.08 | 318.75 | Organic acids and derivatives | down |
| M89T221_3 | Dl-lactate | 89.02 | 221.29 | Organic acids and derivatives | down |
| M115T26 | Alpha-ketoisovaleric acid | 115.02 | 25.53 | Organic acids and derivatives | down |
| M87T130_2 | Pyruvate | 87.01 | 130.01 | Organic acids and derivatives | down |
| M192T301 | Lontrel | 191.98 | 300.57 | Organoheterocyclic compounds | down |
| M330T376 | Camp | 330.07 | 375.61 | Nucleosides, nucleotides, and analogues | down |
| M212T28 | Indoxyl sulfate | 212.00 | 27.70 | Organic acids and derivatives | down |
| M104T286 | N-methyl-l-alanine | 104.07 | 286.37 | Organic acids and derivatives | down |
| M187T25 | 1-hydroxy-2-naphthoic acid | 187.04 | 25.49 | Benzenoids | down |
| M221T219 | Aminomethylphosphonic acid | 221.01 | 219.04 | Organic acids and derivatives | down |
| M116T296_2 | DL-arginine | 116.07 | 295.78 | Organic acids and derivatives | down |
| M173T25 | Isocitric acid | 172.99 | 24.60 | Organic acids and derivatives | down |
| M611T472 | Glutathione, oxidized | 611.15 | 471.54 | Organic acids and derivatives | down |
| M189T24 | Epoxytricarballylic acid | 188.99 | 23.99 | Organic acids and derivatives | down |
| **Differential metabolites in HE group** | | | | | |
| 131.04618 | DL-asparagine | 131.05 | 356.31 | Organic acids and derivatives | up |
| M113T290 | D-galacturonic acid | 113.02 | 290.01 | Organic oxygen compounds | up |
| M104T259 | Glycerophosphocholine | 104.11 | 258.66 | Lipids and lipid-like molecules | up |
| M185T352 | 3-hydroxy-3-methylglutaric acid | 185.03 | 351.76 | Lipids and lipid-like molecules | up |
| M180T68 | 4,6-dinitro-o-cresol | 180.00 | 68.32 | Benzenoids | up |
| M112T231 | Cytosine | 112.05 | 230.68 | Organoheterocyclic compounds | up |
| M147T352 | D-glutamine | 147.08 | 351.64 | Organic acids and derivatives | up |
| M150T268 | L-methionine | 150.06 | 267.82 | Organic acids and derivatives | up |
| M127T352 | Dihydrothymine | 127.05 | 352.41 | Organoheterocyclic compounds | up |
| M207T352 | Pyridate | 207.01 | 351.71 | Organoheterocyclic compounds | up |
| M251T30 | Deoxyinosine | 251.10 | 29.61 | Nucleosides, nucleotides, and analogues | up |
| M145T352 | DL-glutamine | 145.06 | 352.39 | Organic acids and derivatives | up |
| M148T349 | O-acetyl-l-serine | 148.04 | 348.94 | Organic acids and derivatives | up |
| M247T380 | Thr-Glu | 247.09 | 380.42 | Organic acids and derivatives | up |
| M169T352 | Ethyl 2,4-dihydroxy-6-methylbenzoate | 169.06 | 351.67 | Benzenoids | up |
| M199T170 | 5-sulfosalicylic acid | 198.95 | 169.84 | Benzenoids | down |
| M748T78 | 1-palmitoyl-2-oleoyl-phosphatidylglycerol | 747.52 | 77.77 | Lipids and lipid-like molecules | down |
| M205T31 | 3,5-di-tert-butyl-4-hydroxybenzoic acid | 205.16 | 31.34 | Benzenoids | down |
| M263T175 | 2-cis-4-trans-abscisic acid | 263.14 | 174.81 | Lipids and lipid-like molecules | down |
| M141T336 | Kojic acid | 141.02 | 336.46 | Organoheterocyclic compounds | down |
| M105T336 | DL-cysteine | 104.99 | 336.29 | Organic acids and derivatives | down |
| M391T336 | 1-.beta.-d-arabinofuranosyluracil 5'-monophosphate | 390.99 | 336.29 | Nucleosides, nucleotides, and analogues | down |


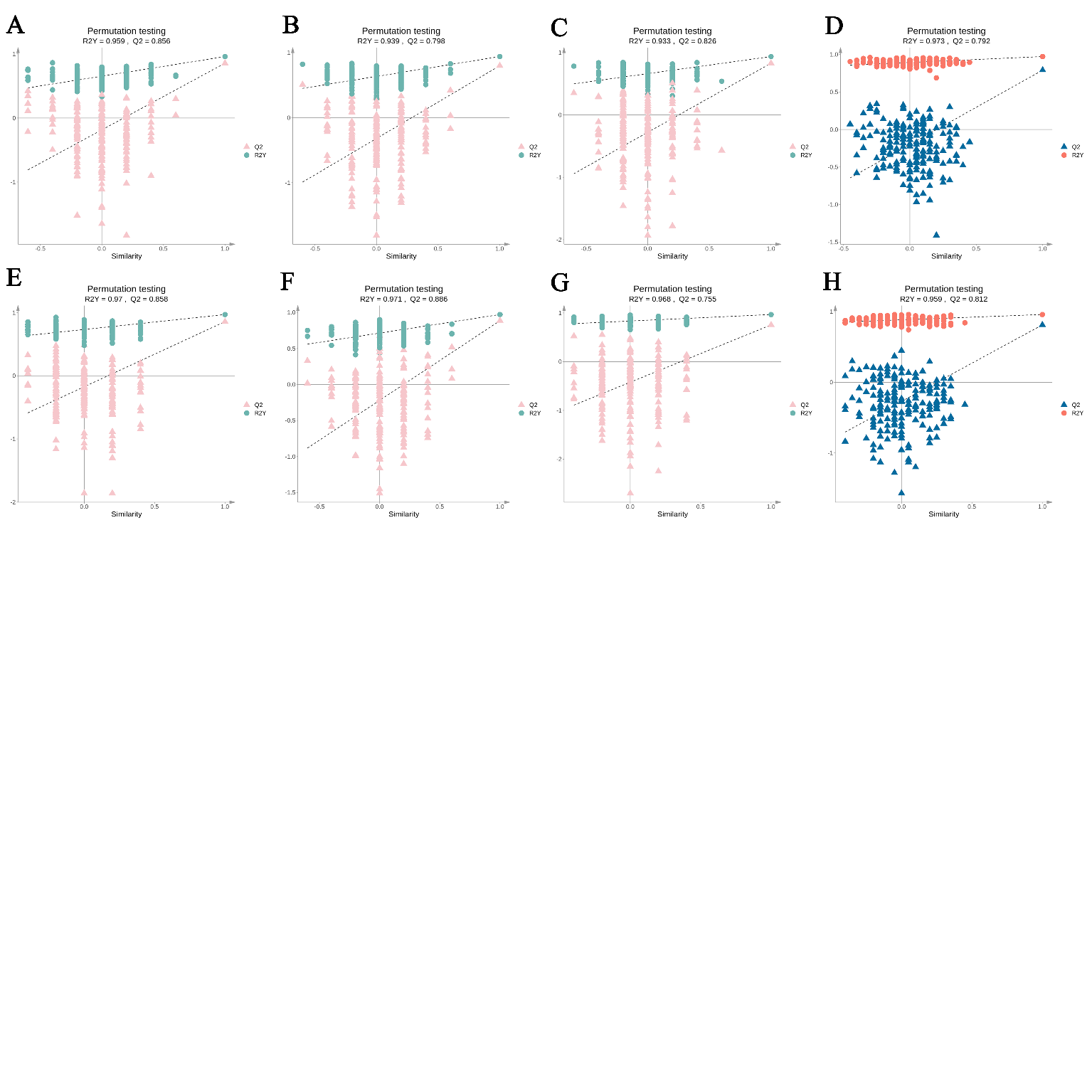


Figure S1. The permutation test of OPLS-DA in the positive ion detection mode: HE vs LE (A), HE vs ME (B), ME vs LE (C), HE vs ME vs LE (D) comparisons. The permutation test of OPLS-DA in the negative ion detection mode: HE vs LE (A), HE vs ME (B), ME vs LE (C), HE vs ME vs LE (D) comparisons.
